# Childhood chronic anterior uveitis associated with vernal keratoconjunctivitis (VKC): successful treatment with topical tacrolimus. Case series

**DOI:** 10.1186/1546-0096-9-34

**Published:** 2011-11-02

**Authors:** Andrea Taddio, Rolando Cimaz, Roberto Caputo, Cinzia de Libero, Laura Di Grande, Gabriele Simonini, Francesca Mori, Elio Novembre, Neri Pucci

**Affiliations:** 1 Institute for Maternal and Child Health - IRCCS "Burlo Garofolo" - Trieste and University of Trieste, Via dell'Istria 65/1, 34100. Trieste, Italy; 2 Rheumatology Unit, Anna Meyer Children's Hospital, Department of Pediatrics, University of Florence, Viale Pieraccini, 24 Careggi, 50134. Florence, Italy; 3 Clinical Ophthalmology Unit, Anna Meyer Children's Hospital, Department of Pediatrics, University of Florence, Viale Pieraccini, 24 Careggi, 50134. Florence, Italy; 4 Allergy and Clinical Immunology Unit, Anna Meyer Children's Hospital, Department of Pediatrics, University of Florence, Viale Pieraccini, 24 Careggi, 50134. Florence, Italy

**Keywords:** Vernal keratoconjunctivitis, Anterior uveitis, treatment, Tacrolimus, children

## Abstract

Uveitis treatment involves topical corticosteroids along with cycloplegic-mydriatics. Particularly severe cases may require systemic corticosteroids and immunosuppressive drugs. Vernal keratoconjunctivitis (VKC) treatment consists of a brief period of topical corticosteroids and/or cyclosporine. In patients refractory to traditional treatment, the use of 0.1% topical ophtalmic FK- 506 (tacrolimus) ointment has been occasionally reported.

This is the first report of the coexistence of uveitis and VKC. The documented response to topical tacrolimus eyedrop of uveitis and VKC is also of interest, in particular since to our knowledge there are no published reports on its clinical use in uveitis.

## Background

Uveitis is an inflammation of the uveal tract or of the adjacent ocular structures. Commonly uveitis is idiopathic, but identifiable causes have been reported as well (e.g. infections; systemic diseases). These systemic illnesses are usually driven by autoimmune mechanisms, particularly in those children with connective tissue involvement [1]. Juvenile idiopathic arthritis (JIA) is commonly associated with chronic iridocyclitis in children [2], particularly in those with oligoarticular onset and antinuclear antibody (ANA) positivity. Unlike most forms of anterior uveitis, the onset of chronic uveitis in JIA patients tends to be insidious and entirely asymptomatic, although later in the course of the disease, some children may develop symptoms attributable to uveitis (pain, redness, headache, photophobia, vision changes). JIA-associated uveitis typically affects girls and is bilateral. Recurrences are frequent and an active state of inflammation may alternate between eyes [2]. Several other autoimmune conditions may involve the uvea: spondyloarthropathies, sarcoidosis, Behçet's syndrome, Vogt- Koyanagi-Harada (VKH) syndrome, vasculitides such as Wegener's granulomatosis and Kawasaki disease and inflammatory bowel diseases.

The active phase of inflammation usually requires topical corticosteroids along with cycloplegic- mydriatics. Systemic corticosteroids and immunosuppressive drugs have often been employed for the severe or chronic patterns of the disease [3]. In the last 10 years, anti-TNF drugs have been successfully utilized for the treatment of uveitis and have become a useful tool for clinicians [4]. Vernal keratoconjunctivitis (VKC) is a chronic, bilateral inflammation of the superior tarsal and limbar palpebral conjunctiva. The onset typically occurs between 3 and 25 years of age. The most common symptoms are itching, photophobia, burning, and lacrimation. The clinical signs consist of large conjunctival papillae on the back of the superior tarsus, raised Horner-Trantas dots (gelatinous, white clumps of degenerated eosinophils usually located at the superior limbus), areas of superficial punctate keratitis and, in severe cases, well-demarcated, sterile corneal shield ulcers, superiorly located [5].

VKC may be somehow linked to atopic conditions. In fact, a positive response to skin and Radio-Allergo-Sorbent Test (RAST) was found in 57% and 52% of VKC patients, respectively [5]. Moreover, total serum IgE levels have been shown to be elevated [6] and local production of IgE in tears has also been postulated [7]. The pathogenesis remains still unknown but a T helper (Th)2- driven mechanism with mast cell, eosinophilic, lymphocytic involvement together with the production of both regulatory and inflammatory cytokines such as IL-4, IL-5, IL-13, has also been postulated [8]. The treatment consists of topical corticosteroids that should be carefully administered just for short periods, in order to avoid a possible secondary development of glaucoma and/or cataract [9]. A 1-2% solution of topical cyclosporine eyedrops can be considered as an alternative [10]. In patients refractory to traditional treatments, 0.1% topical ophthalmic FK-506 (tacrolimus) eyedrops have been tried [11]. Tacrolimus is a macrolide antibiotic with potent immunosuppressive activity. It belongs to the calcineurin-inhibitor family and has immunosuppressive activity similar to cyclosporine.

Uveitis and VKC seem to arise from two different, evenly opposed immunologic pathways: on one side Th1 cells, that are known to be involved in the pathogenesis of organ-specific autoimmune disorders; on the other a Th2 response which is responsible for atopic disorders in genetically susceptible individuals. Both VKC and uveitis can be considered rare diseases. The incidence of VKC was reported to be 1/100,000/year, with a higher rate in males under 16 years of age (10/100,000). It has a prevalence of 7.8/100,000, again with a higher rate in young males (57/100,000) [12]. Uveitis incidence varies between 17 and 27 new cases per 100,000 people per year. In children uveitis rates are lower with a variable annual incidence from 4.3 to 6.9 per 100,000 and a prevalence of 30 cases/100,000 [13].

We report here the unusual association between uveitis and vernal keratoconjunctivitis in three children referred to A. Meyer Children's Hospital in Florence, Italy, as well as the encouraging response of both uveitis and VKC to 0.1% topical FK-506 (tacrolimus) eyedrops application. Such a medication has been prepared by Prograf. One ml = 5 mg vial of tacrolimus (Astellas Pharma Spa) is diluted with 4 ml of Polyvinil alchol, Hypotears (Novartis Pharma Spa).

## Case Presentations

### Case 1

**Figure 1:**
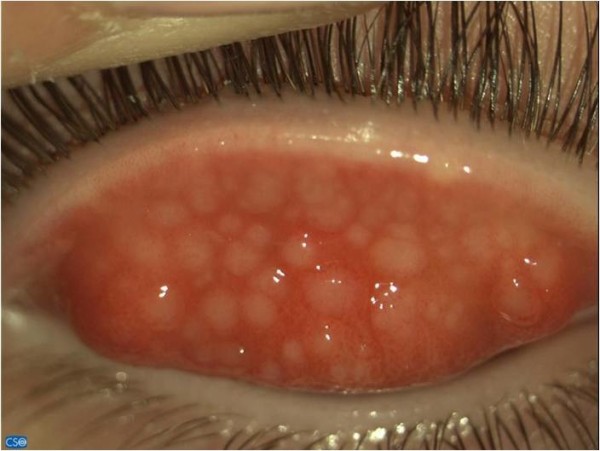
**Giant tarsal follicular conjunctivitis in a patient with Vernal Keratoconjunctivitis (VKC)**.

After the diagnosis of ANA-positive polyarticular JIA, a 3-year-old female started to be treated with non-steroidal anti-inflammatory drugs (NSAIDS, ibuprofen 10 mg/kg/dose 3 times a day), methotrexate (15 mg/m^2^/subcutaneously once a week) and later with etanercept (anti-TNF soluble receptor antagonist; dosage: 0.8 mg/kg subcutaneously once a week) with good clinical control of arthritis. She was 5 years old, still on etanercept and methotrexate, when uveitis was diagnosed. Her exam showed the presence of keratic precipitates and a positive Tyndall sign (++) with cell grading of 3 on slit lamp exam. Her etanercept was stopped. The patient was started on topical steroids (dexamethasone: 2 drops per eye three times a day) and mydriatic drops (1 drop per eye once a day). The uveitis responded to drops and methotrexate. Unfortunately the methotrexate had to be stopped after 3 months due to liver enzyme elevation. As a consequence, relapses did persist almost three times a year, requiring repeated topical steroid courses. Three years later during the spring, she started to complain of itching and photophobia. The ophthalmologic examination revealed clinical giant tarsal follicular conjunctivitis with Horner-Trantas dots (Figure 1), leading to the diagnosis of VKC. Topical 1% cyclosporine A was started without improvement. During such a treatment regimen, uveitis flares frequently occurred every three months, requiring the employment of topical steroids to obtain a good control of both VKC and uveitis. Because of the persistence of VKC and the recurrences of uveitis, 0.1% topical ophthalmic FK-506 (tacrolimus) eyedrops were started (1 drop per eye three time a day). VKC relief was prompt, and interestingly, her uveitis also resolved. Treatment with topical tacrolimus was continued for three months. then it was tapered and finally stopped after six months from its introduction. During the following 10 months of follow-up since it was completely withdrawn, no new uveitis flares occurred.

### Case 2

A 3 year-old female was referred to our Rheumatology Unit for an 8-week history of knee effusion. Physical examination was unremarkable except for the presence of left knee arthritis. Laboratory findings were all normal except for anti-nuclear antibodies positivity. The recent history, the clinical features and the presence of asymptomatic uveitis (keratic precipitates and positive Tyndall sign ++, and a cell grading of 3 in the left eye were consistent with oligoarticular JIA with uveitis. After an unsuccessful trial with NSAIDS (ibuprofen 10 mg/kg/dose 3 times a day), methotrexate was promptly started (15 m^2^/subcutaneously once a week) with excellent improvement of the arthritis. Unfortunately, uveitis rapidly affected both eyes and frequently flared (almost every three months) requiring topical steroids (dexamethasone: 2 drops per eye three times a day) and cyclopegic drugs (1 drop per eye once a day). One year later, during a phase of uveitis quiescence, she started to complain of photophobia and itching. Giant tarsal papillar conjunctivitis and Horner-Trantas dots were found, allowing the diagnosis of VKC which was successfully treated with topical 1% cyclosporine A (1 drop per eye twice a day). Unfortunately, because of burning on application of the medication, the patient stopped the cyclosporine two months later. The VKC then flared again.

Due to the flare of the VKC and the uveitis, 0.1% topical ophthalmic FK-506 (1 drop per eye three time a day) was started one month later. The VKC rapidly improved, and surprisingly, the uveitis completely resolved in a few days. Topical tacrolimus was continued at full dosage for the first three months and then tapered and stopped within 5 months from its introduction. For the next 12 months, no flares of the VKC or uveitis occurred.

### Case 3

A 7 year-old female was referred to our Ophthalmologic Unit because of a two year history of ocular burning and lacrimation with photophobia, usually worse from March to October. Clinical signs of VKC were found (giant limbar papillar conjunctivitis and Horner-Trantas dots). Slit lamp exam also detected keratic precipitates and a positive Tyndall sign (cell grading: 2), consistant with bilateral uveitis. No infections and/or systemic associated diseases were detected and the diagnosis of idiopathic uveitis was made. VKC was successfully controlled with topical 1% cyclosporin A (1 drop per eye twice a day), but frequent uveitis relapses required several topical steroid courses (dexamethasone: 2 drops per eye three times a day) and cyclopegic drugs (1 drop per eye once a day). For this reason and on the basis of the previous two cases, 0.1% topical treatment with FK-506 eyedrops was started (1 drop per eye three time a day), with a prompt recovery of both the diseases within 10 days. Topical tacrolimus was maintained at full dosage for three months before tapering and then discontinued after six months. VKC and uveitis were still in remission off therapy after a follow-up of one year. No sign or symptom compatible with a rheumatic disease later appeared.

## Discussion

We have described three children with coexisting uveitis and VKC, who responded well to topical tacrolimus for both conditions. The treatment regimen used for the three patients (two children had JIA-related uveitis, the third child suffered from an idiopathic form) was the same. At the time of starting tacrolimus eye drops, no other topical drugs had been recently utilized and children receiving systemic treatment for JIA continued with the same regimen. No child was simultaneously taking systemic or topical corticosteroids while on topical tacrolimus. The drug was prepared by Prograf^® ^and was administered three times a day (one drop per eye). The dilutions remained stable for a month if kept at 4°C. All patients received the full dosage for the first three months, then the tacrolimus was tapered until complete cessation within six months of starting the tacrolimus.

Our current clinical practice for chronic uveitis management includes the use of topical corticosteroids along with cycloplegic-mydriatics initially. Immunosuppressant drugs such as methotrexate and cyclosporine A and eventually anti-TNF drugs are used for resistant cases. There has been no data about the use of topical tacrolimus in anterior uveitis. Yet since the morbidity of chronic uveitis is high and systemic drugs may cause severe side effects, topical tacrolimus might be a valid alternative for mild-moderate recurrent uveitis flares. Severe recurrent flares may need more aggressive regimen options.

To our knowledge, topical tacrolimus has been used by intravitreal injection in animal models, but no reports have been published on its use for human anterior uveitis [14]. There have been reports on tacrolimus treatment for immune posterior uveitis. Posterior uveitis likely reflects different pathogenetic pathways and is seen in other conditions, like Behcet's disease or Crohn's disease [15,16]. In these reports systemic tacrolimus was given with good efficacy on uveitis control and excellent safety profile.

Further studies on larger cohorts of patients are needed to evaluate the efficacy and the safety of topical tacrolimus for the treatment of anterior uveitis. Moreover, the employment of topical tacrolimus may expose the patient to a higher risk of herpes keratitis, but so far this kind of complication has not been described. In the three cases reported, only mild discomfort was associated with its application (even at the concentration of 0.1% which is higher than the 0.03% described in the literature) such as a temporary sense of burning during the very first days of therapy.

There appears to be a low rate of tacrolimus systemic adsorption when administered in the eyes of rabbits even at a concentration 10 times higher than the one we used [17]. According to our experience, no clinical side effects were observed. Every three months liver and renal functions were tested and always found normal, and serum tacrolimus levels always remained within the normal range.

In all 3 cases described, uveitis frequently recurred, requiring several courses of topical steroids. In the two cases with JIA-related uveitis, systemic treatment (methotrexate alone or with anti-TNF antagonist receptor) was useful for control of the arthritis but did not show any relevant clinical effects on eye symptoms. It is important to point out that JIA-associated uveitis is known to have relapses and it is expected to have an asymmetric course. A one year follow-up period might not be considered sufficient. During this one year period our patients underwent two monthly ophthalmologic evaluations that did not reveal signs attributable to uveitis. However, none of our patients had experienced such a long uveitis-free period in the past, supporting the clinical benefits of tacrolimus. In our first patient, uveitis developed while on therapy with etanercept. There have been reports of etanercept itself triggering uveitis [4]. However, etanercept was withdrawn with no significant amelioration of the uveitis.

To our knowledge, this is the first report describing the association between an allergic chronic ocular disease such as VKC with an autoimmune disease such as uveitis.

Recent reports have shown that the Th1-proinflammatory cytokine IFN-γ, and the Th2, IL-4, IL-13 cytokines, may coexist in both inflammatory and allergic responses [12]. Other experimental studies have challenged the Th1-Th2 balance paradigm in both allergic diseases and in uveitis [18].

In our hospital, we follow 408 patients affected by VKC and 99 children with uveitis. Considering that three patients had both diseases, we can assume that the prevalence of uveitis in VKC patients is much higher (0.7%) than in controls (0.02%). Similarly, VKC seems to be more frequently associated with uveitis, with a prevalence of 3%. These considerations make it unlikely that the coexistence of the two diseases in the same patient could be considered a mere coincidence, even if the explanation of the relationship is still unknown.

## Conclusion

We report 3 children who simultaneously developed VKC and anterior uveitis, 2 who had JIA and one child with idiopathic uveitis. All 3 children failed conventional topical and systemic treatment for their eye disease before the use of topical tacrolimus appeared to put both eye diseases in remission for at least one year. The use of topical tacrolimus for severe eye problems such as resistant VKC and uveitis should be explored further.

## Consent

Written informed consent was obtained from the patients for publication of this case report and any accompanying images. A copy of the written consent is available for review by the Editor-in-Chief of this journal

## List of abbreviations

JIA: Juvenile Idiopathic Arthritis;ANA: Antinuclear antibody;VKC: Vernal keratoconjunctivitis.

## Competing interests

The authors declare that they have no competing interests.

## Authors' contributions

AT has made substantial contributions to conception and was involved in the drafting of the article.

RC has made substantial contributions to conception and interpretation of data as well as revision the manuscrpt for important intellectual content. CL, LG, FM, GS, EN and NP have made substantial contributions in acquisition of data and were involved in drafting the article. All authors read and approved the final manuscript.

## References

[B1] Heinz C Mingels A Goebel C Fuchsluger T Heiligenhaus A Chronic uveitis in children with and without juvenile idiopathic arthritis: differences in patient characteristics and clinical course J Rheumatol 2008 35 1403 7 18484686

[B2] Cassidy JT Sullivan DB Petty RE Clinical Patterns of chronic iridocyclitis in children with juvenile rheumatoid arthritis Arthritis Rheum 1977 20 224 227 318121

[B3] Sobrin L Christen W Foster CS Mycophenolate mofetil after methotrexate failure or intolerance in the treatment of scleritis and uveitis Ophthalmology 2008 115 1416 21 10.1016/j.ophtha.2007.12.011 18221998

[B4] Simonini G Zannin ME Caputo R Falcini F de Martino M Zulian F Cimaz R Loss of efficacy during long term infliximab therapy for sight-threatening childhood uveitis Rheumatology (Oxford) 2008 47 1510 4 10.1093/rheumatology/ken298 18676502

[B5] Bonini S Bonini S Lambiase A Marchi S Pasqualetti P Zuccaro O Rama P Magrini L Juhas T Bucci MG Vernal keratoconjunctivitis revisited: a case series of 195 patients with long-term followup Ophthalmology 2000 107 1157 1163 10.1016/s0161-6420(00)00092-0 10857837

[B6] Fujishima H Saito I Takeuchi T Tsubota K Immunological characteristics of patients with vernal keratoconjunctivitis Jpn J Ophthalmol 2002 46 244 8 10.1016/s0021-5155(02)00481-1 12063032

[B7] Aalders-Deenstra V Kok PT Bruynzeel PL Measurement of total IgE antibody levels in lacrimal fluid of patients suffering from atopic and non-atopic eye disorders. Evidence for local IgE production in atopic eye disorders? Br J Ophthalmol 1985 69 380 384 10.1136/bjo.69.5.380 PMC1040606 3994956

[B8] Bonini S Bonini S IgE and non-IgE mechanisms in ocular allergy Ann Allergy 1993 71 296 299 8373003

[B9] Tabbara KF Ocular complications of vernal keratoconjunctivitis Can J Ophthalmol 1999 34 88 92 10321319

[B10] Pucci N Novembre E Cianferoni A Lombardi E Bernardini R Caputo R Campa L Vierucci A Efficacy and safety of cyclosporine eyedrops in vernal keratoconjunctivitis Ann Allergy Asthma Immunol 2002 89 298 303 10.1016/S1081-1206(10)61958-8 12269651

[B11] Joseph MA Kaufman HE Insler M Topical tacrolimus ointment for treatment of refractory anterior segment inflammatory disorders Cornea 2005 24 417 20 10.1097/01.ico.0000151507.49565.6e 15829797

[B12] Gor DO Rose NR Greenspan NS TH1-TH2: a procrustean paradigm Nat Immunol 2003 4 503 505 10.1038/ni0603-503 12774069

[B13] Leonardi A Busca F Motterle L Cavarzeran F Fregona IA Plebani M Secchi AG Case series of 406 vernal keratoconjunctivitis patients: a demographic and epidemiological study Acta Ophtalmol Scand 2006 84 406 10 10.1111/j.1600-0420.2005.00622.x 16704708

[B14] Oh-i K Keino H Goto H Yamakawa N Murase K Usui Y Kezuka T Sakai J Takeuchi M Usui M Intravitreal injection of Tacrolimus (FK506) suppresses ongoing experimental autoimmune uveoretinitis in rats Br J Ophthalmol 2007 91 237 42 10.1136/bjo.2006.103168 PMC1857637 16987901

[B15] Figueroa MS Ciancas E Orte L Long-term follow-up of tacrolimus treatment in immune posterior uveitis Eur J Ophthalmol 2007 17 69 74 10.1177/112067210701700110 17294385

[B16] Hogan AC McAvoy CE Dick AD Lee RW Long-term efficacy and tolerance of tacrolimus for the treatment of uveitis Ophthalmology 2007 114 1000 6 10.1016/j.ophtha.2007.01.026 17467532

[B17] Fujita E Teramura Y Mitsugi K Ninomiya S Iwatsubo T Kawamura A Kamimura H Absorption, distribution, and excretion of 14C- labeled tacrolimus (FK506) after a single or repeated ocular instillation in rabbits J Ocul Pharmacol Ther 2008 24 333 43 10.1089/jop.2007.0086 18476802

[B18] Randolph DA Stephens R Carruthers CJ Chaplin DD Cooperation between Th1 and Th2 cells in a murine model of eosinophilic inflammation J Clin Invest 1999 104 1021 1029 10.1172/JCI7631 PMC408580 10525040

